# Adaptive Exposure Control for Line-Structured Light Sensors Based on Global Grayscale Statistics

**DOI:** 10.3390/s25041195

**Published:** 2025-02-16

**Authors:** Yuehua Li, Qingfeng Zhao, Po Hu, Hao Zhang, Ziheng Zhang, Xiaohong Liu, Jingbo Zhou

**Affiliations:** 1 School of Mechanical Engineering, Hebei University of Science and Technology, Shijiazhuang 050018, China; yuehua.hrbin@163.com (Y.L.); zhaoqqf973@126.com (Q.Z.); zh99150@outlook.com (H.Z.); 18033857268@163.com (Z.Z.); liuxh@hebust.edu.cn (X.L.); 2 School of Mechanical Engineering, Shijiazhuang Tiedao University, Shijiazhuang 050043, China; hupo@stdu.edu.cn

**Keywords:** line-structured light sensor, quality evaluation of stripe images, global grayscale statistics, adaptive exposure control

## Abstract

Stripe images are crucial for ensuring the measurement quality of line-structured light sensors. To improve the measurement effectiveness of objects with different shapes, materials, and colors, an adaptive exposure method is proposed based on global grayscale statistical analysis of stripe images. The logarithm sum of grayscale statistical results is calculated as the quality evaluation parameter for each stripe image. Theoretical analysis and experiments demonstrate that the proposed quality evaluation value exhibits an approximate linear relationship with a camera’s exposure time. Subsequently, an adaptive exposure control method is developed. The influence of control system parameters on measurement results is also analyzed in detail. The experimental results show that our method can adaptively adjust a camera’s exposure time according to different surface characteristics. Both the number of effective measurement points and the accuracy are improved.

## 1. Introduction

Line-structured light sensors (LSLSs) mainly comprise a camera and a laser line projector. In the measurement process, a laser line is projected onto the object, and the camera captures perturbed stripe images. A two-dimensional (2D) profile can be calculated according to the pixel coordinates of the laser stripe, the camera’s intrinsic parameters, and the equation of the laser plane [1]. LSLSs have the advantages of having a simple structure, fast measurement speed, and high accuracy. They have broad application prospects in various fields, such as three-dimensional (3D) measurement [2], quality inspection [3], and character recognition [4].

Currently, research on LSLSs mainly focuses on the center extraction of stripe images [5], sensor calibration [6], and the integration of sensors with other motion coordinate systems for different measurement applications [7,8]. Once the sensor has been calibrated, the camera’s intrinsic parameters, such as the distortion parameters and the relative position between the camera and laser line projector, are kept unchanged. However, stripe images are always changing according to the geometry, material, color, and surface roughness of the object being measured. The quality of stripe images affects the accuracy and reliability of the center extraction results, thereby influencing the final measurement results. Ensuring stripe image quality with adaptive control is the key to achieving high-precision results and is a core issue in LSLS research [9].

Most research on the adaptive control of structured light measurements focuses on fringe projection systems based on digital light processing (DLP) [10]. With DLP devices, flexible fringe patterns can be generated, and the intensity can be adjusted even at the pixel level [11,12]. Adaptive control can be achieved by automatically adjusting the camera exposure time and fusing the fringe images [13,14,15]. Other strategies include generating optimal fringe projection patterns via surface segmentation [16], the analysis of saturated pixels in captured images [17], and the application of intensity coefficient templates [18], misaligned gray code [19], local surface reflectivity [20], and accurate optical models [21]. To enhance the measurement efficiency of highly reflective surfaces, adaptive projection can be combined with a curve-fitting algorithm [22] or the original and inverse projection method [23]. All of these methods enable measurement flexibility and facilitate the implementation of structured light methods. In contrast to DLP-based 3D measurement techniques, LSLSs feature a simpler design and lower cost. The laser line is emitted from a semiconductor laser source and shaped by a Powell lens. Its brightness can be adjusted only as a whole. Additionally, the sensor keeps moving during the scanning process, rendering DLP-based adaptive control methods unsuitable for LSLSs.

Variations in color, material, and roughness directly impact the quality of stripe images. To address issues such as reflection interference, overexposure, and underexposure, different methods have been developed to enhance the accuracy of stripe center extraction, like the mass–spring method [24], the stripe segmentation method [25], and the multi-scale analysis method [26]. However, these methods are often intricate and time consuming. Enhancing the quality of stripe images themselves is a more effective approach to improving measurement results. Song et al. [27] employed wavelet decomposition to adjust the brightness of stripe images by regulating the input voltage of the laser projector. Tang et al. [28] utilized dual-tree complex wavelet transform to control laser intensity. It is important to note that wavelet technology presents substantial computational challenges, and the aforementioned wavelet-based methods are solely designed for selecting an optimal intensity, thereby lacking real-time control capabilities. In a separate effort, Li et al. [29] devised an imaging system for laser scanners using a silicon-based reflective liquid crystal (LCoS) device and explored optical image adaptation methods for rectifying local oversaturation. The LCoS device effectively attenuates the light reaching the image pixels, thus avoiding overexposure. However, this method requires additional hardware such as optical devices, the LCoS device, and control units. It also introduces more complex calibration processes. Zhou et al. [30] achieved adaptive sensor control by modulating the camera exposure time to make the average stripe width within a desired range. The computation of stripe width is time consuming, however, and hinders the increase in sampling frequency.

The width of the stripe directly affects the uncertainty of center extraction. Typically, when the stripe width is between 8 and 10 pixels, optimal uncertainty values can be achieved [31]. However, width computation is time consuming and may not meet real-time adaptive control requirements. It is known that the grayscale variation of laser stripes directly relates to the grayscale distribution curves of stripe images. Our research aims to derive a quality evaluation parameter from the grayscale distribution curve. By adjusting the camera’s exposure time, stripe quality can be maintained within an ideal range. This, in turn, enhances the integrity and accuracy of the measurement results.

## 2. Measurement Principle

### 2.1. Measurement Principle of LSLS

**Figure 1:**
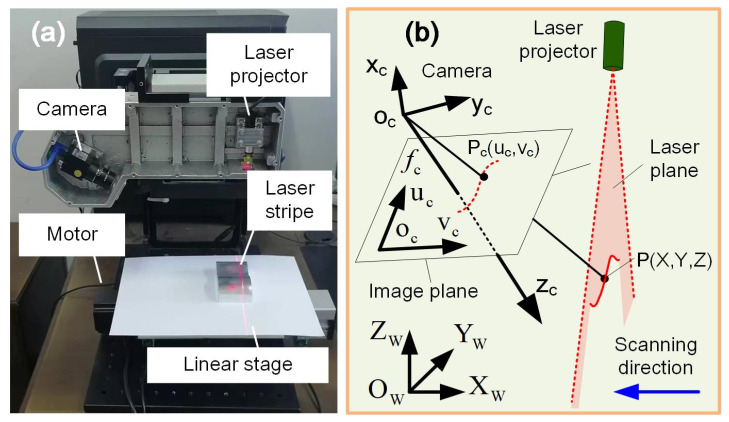
Line-structured light measurement system. (**a**) System components. (**b**) Measurement principle.

As shown in Figure 1a, the line-structured light measurement system (LSLMS) mainly consists of a camera (MV-SUA133GM, MindVision Technology Co. Ltd., Shenzhen, China), a laser line projector (Shengzuan Technology Co. Ltd., Shenzhen, China), and a linear stage. The laser line is projected onto the part, and the camera captures stripe images perturbed by the surface. Based on the pixel coordinates of the stripe center, the camera’s intrinsic parameters, and the equation of laser plane, the corresponding profile can be solved. As the linear stage moves, a series of profiles can be obtained. With the pre-calibrated relative relationship between sensor and motion direction, 3D results can be achieved.

The measurement principle is illustrated in Figure 1b. O_W_X_W_Y_W_Z_W_ and o_c_x_c_y_c_z_c_ are the world and the camera coordinate systems, respectively. o_c_u_c_v_c_ is the pixel coordinate system with o_c_u_c_//o_c_x_c_, o_c_v_c_//o_c_y_c_. o_c_x_c_ is the optical axis, and *f_c_* is the focal length of the lens. O_W_X_W_Y_W_Z_W_ is positioned on the top surface of the linear stage with the O_W_X_W_ axis aligned with the scanning direction. This would make the surface reconstruction more convenient. Here, we mainly focus on the adaptive control of the LSLMS.

### 2.2. Evaluation of Stripe Images

The quality evaluation of stripe images serves as a fundamental prerequisite for the implementation of adaptive control. Among the various techniques of image analysis, the grayscale histogram is one of the most commonly used methods. Grayscale statistical values of stripe images can be expressed as *H*(*I*) = *n_I_*, where *I* represents the grayscale values, *I* = 0, 1, 2, …, 255, and *n_I_* is the number of pixels with the grayscale value *I*. In the measuring process, the total number of pixels remains unchanged. Thus, normalization is not necessary in this area of research.

**Figure 2:**
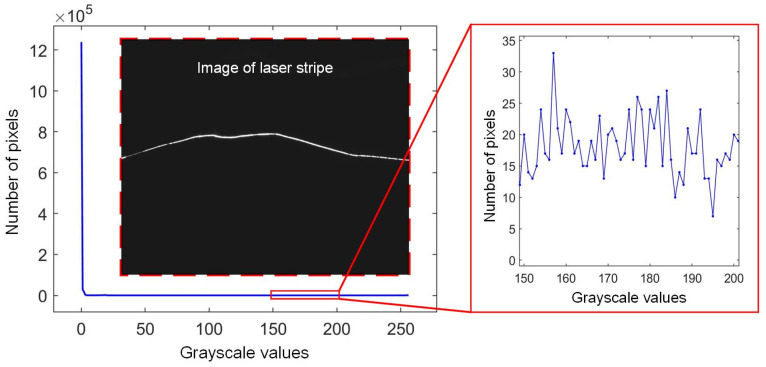
Image of laser stripe and its corresponding histogram curve.

Figure 2 shows a stripe image and its corresponding histogram curve. Unlike the common visual inspection images, it can be seen that only the pixels within the stripe area are highlighted. These pixels account for only a minuscule proportion of the entire image, while the majority of pixels have a grayscale value of 0. For our camera, it has 1280 × 1024 pixels. During the measurement process, the number of pixels with a grayscale value of 0 can reach up to 1.2 × 10^6^. The number of pixels with other grayscale values is extremely small, resulting in an “L” shape in the histogram curve. In this scenario, the variation in this curve is hardly visible when the camera exposure time is changed. Therefore, this histogram curve cannot be directly utilized for adaptive control.

**Figure 3:**
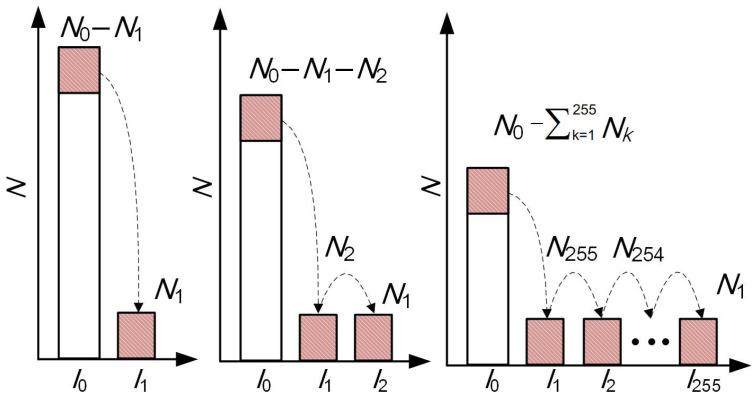
Variation in the number of different grayscale pixels with exposure time.

When a laser stripe is projected onto an object, the reflected light carries the geometric information of the profile. The grayscale value of each pixel, *T*, in the image can be expressed by

(1) $$T=t\cdot \alpha pRs\left(I_{e}+I_{d}+I_{s}\right)+\varepsilon ,$$

where *t* is the exposure time, *α* is the amplification circuit gain, *p* is the charge transfer efficiency, *R* is the sensitivity, *s* is the pixel area, *I_d_* is the diffuse intensity, *I_s_* is the specular intensity, *I_e_* is the reflected intensity of ambient light, and *ε* represents the noise. It can be seen that the grayscale value of the pixel is linearly related to the exposure time. For this special illumination scenario of laser stripes, the number of pixels with different grayscale values varies with the exposure time, as shown in Figure 3.

Due to the narrow and thin characteristics of the laser stripe, the gray value of the pixels on the stripe firstly increases to *I*_1_ when the exposure time increases by Δ*t*. Assuming the number of pixels is *N*_1_ for *I*_1_, the remaining pixels with the grayscale value *I*_0_ changes from the initial value of *N*_0_ to *N*_0_ − *N*_1_. As can be seen from Equation (1), the gray values of all pixels on the stripe increase with the exposure time. When the exposure time increases by another Δ*t*, the gray value of these stripe pixels further increases and “migrates” to *I*_2_. At this time, another *N*_2_ pixel increases from the total pixels to *I*_2_, and so on until *N*_1_ reaches *I*_255_ and is saturated.

The total number of pixels, *N*_0_, does not change along with the exposure time. Assume that the camera exposure time increases uniformly by *λ* times, and the number of migrated pixels is *N_k_*. The number of pixels corresponding to each gray value is logarithmically processed and then summed as

(2) $$f\left(\lambda \Delta t\right)=\log\left(N_{0}-\lambda N_{k}\right)+\lambda \log\left(N_{k}\right),$$

Since *N*_0_ ≈ 1.3 × 10^6^, the number of pixels on the laser stripe, *λN_k_*, is about 1.3 × 10^4^ under normal exposure conditions (the width of the stripe is calculated as 10 pixels). Therefore, log(*N*_0_ − *λN_k_*) ≈ log(*N*_0_), *f*(*λ*Δ*t*) increases approximately linearly with the exposure time. To illustrate the relationship between the gray distribution and the exposure time more clearly, we calculate the logarithmic curve of the gray distribution by Equation (3).

(3) $$Q\left(I\right)=\log\left[H\left(I\right)+1\right],$$

where *H*(*I*) is the gray distribution of the current stripe image plus 1 to avoid singular values. Furthermore, the stripe quality at exposure time *t* is defined as

(4) $$F\left(t\right)=\sum_{I=0}^{255}Q\left(I\right),$$

In this case, the deviation in the quality parameters between the current and the reference stripe is

(5) $$E\left(t\right)=\sum_{I=0}^{255}\left\{\log\left[H\left(I\right)+1\right]-\log\left[\alpha H_{r}\left(I\right)+1\right]\right\},$$

where α is the ratio of the number of effective cross-sections, and *H_r_*(*I*) is the gray distribution of the reference stripe image.

### 2.3. Adaptive Control of Exposure Time

**Figure 4:**
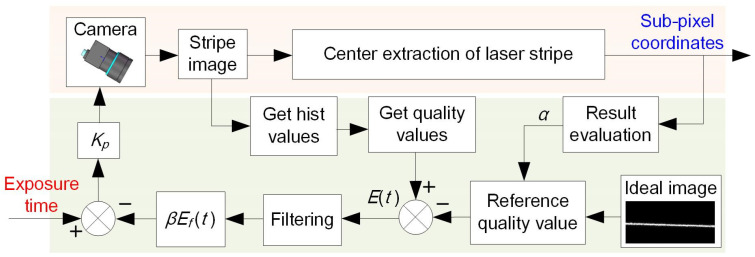
Block diagram of the adaptive control system.

The block diagram of the adaptive control system is shown in Figure 4. When adaptive control is not applied, the measurement process is typically accomplished through the following steps: capturing the stripe images, extracting the stripe center points, and computing the measurement profile according to the camera’s intrinsic parameters and the equation of laser plane. The exposure time of the camera is fixed for the entire scanning process. Nevertheless, the surface characteristics of the object may change during the measurement process, which makes it difficult to ensure the quality of the laser stripe.

Based on the gray distribution curve, the stripe quality value can be calculated in real time according to Equation (4). Simultaneously, the occlusion ratio α of the image and the reference stripe quality value can be calculated according to the center extraction result. By comparing the current stripe quality value with the reference value, the deviation of stripe quality *E*(*t*) can be obtained. Here, *β* is a conversion factor that connects the stripe quality values and camera exposure time, *E_f_*(*t*) is the filtered result of *E*(*t*), and *K_p_* is the proportional gain. When the stripe quality value is consistent with that of the ideal stripe image, the camera achieves the optimal exposure time and a high-quality stripe image can be obtained.

## 3. Experiment and Discussion

### 3.1. Relationship Between Exposure Time and Stripe Quality

**Figure 5:**
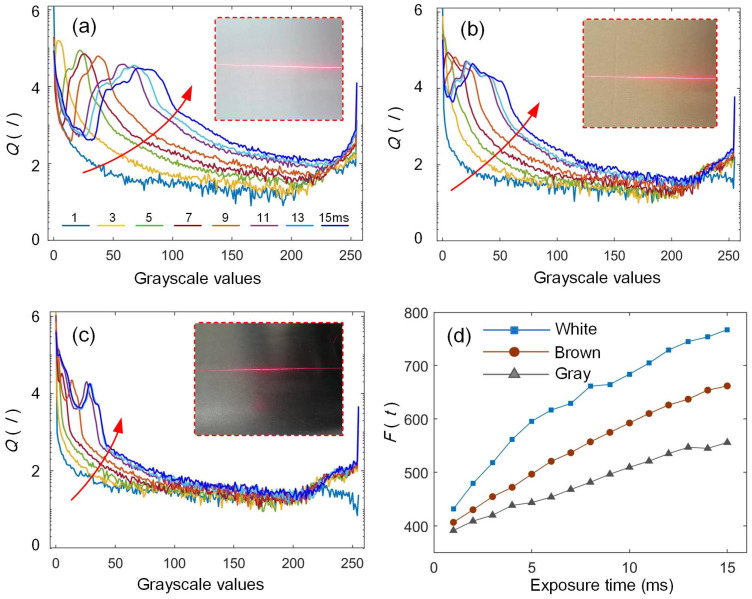
Influence of exposure time on stripe quality. (**a**–**c**) Logarithmic curves of gray distribution on different surfaces at different exposure times, and (**d**) stripe quality curves of different surfaces under different exposure times.

To analyze the influence of exposure time on the quality values of laser stripe, objects with different materials and colors are selected to obtain the logarithmic curves under different exposure times, as shown in Figure 5a–c. For these cases, it can be seen that the number of large gray pixels increases with the exposure time. The gray logarithmic curves move upwards and the area under the curve becomes larger. Thus, the value of image quality *F*(*t*) also increases. The lighter the color of the measured surface, the more significant the upward shift in the curves, and higher-quality values can be obtained by integration. The logarithmic curve reaches its maximum value at zero, which indicates that the gray values of most pixels are zero under normal circumstances. The stripe images are processed using a computer with an Intel i5-3470 CPU and 4GB RAM. The computation time for each stripe quality value is 1.8ms, which has been reduced effectively compared with the width computation method [30].

The corresponding curves, *F*(*t*), can be obtained for the three cases, as shown in Figure 5d. It can be observed that *F*(*t*) exhibits an approximately linear relationship with the exposure time, which is also consistent with the conclusion drawn from the analysis of Equation (2). The exposure time has included all of the cases, from under- to overexposure. This indicates that the stripe quality can be evaluated by Equation (4), and the conversion coefficient *β* between the quality parameter and the camera exposure time can be determined by fitting the curves in Figure 5d. The proposed quality parameters change linearly with the exposure time, which is beneficial to the adaptive control. Although the values of *β* are different for different surface colors, it does not affect the control performance. When the measurement is performed on the brown surface, *β* can be obtained as 0.053 through linear fitting.

### 3.2. Control Performance Analysis

**Figure 6:**
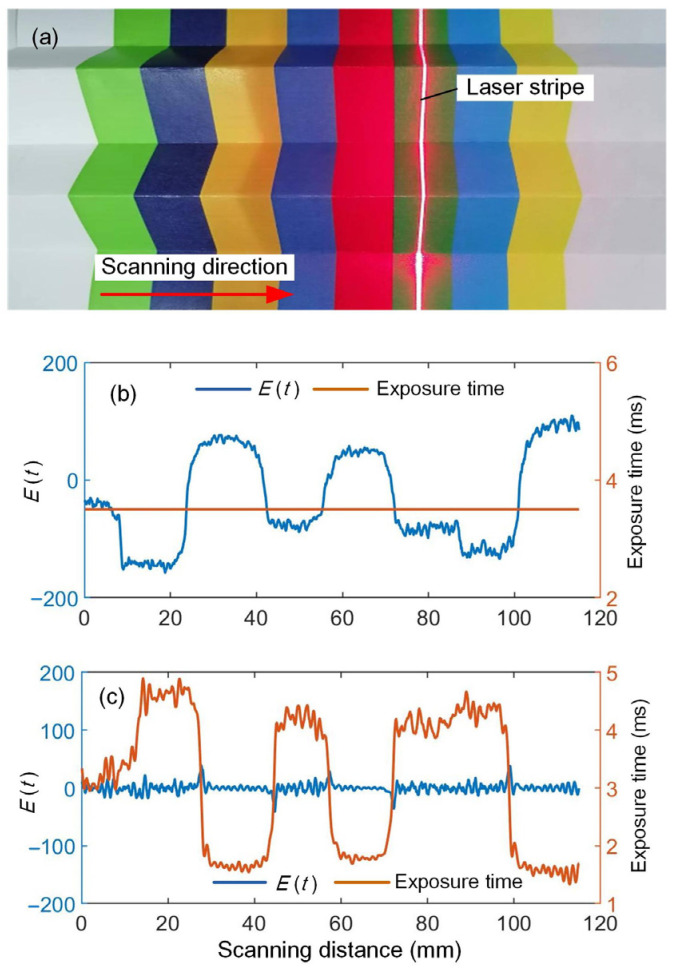
Measurement process without and with adaptive control; (**a**) paper with color stripe, (**b**) without adaptive control, and (**c**) with adaptive control.

To validate the effectiveness of the adaptive control method, color stripes are printed on a piece of paper, and it is then folded into an “M” shape, as shown in Figure 6a. The laser line is carefully adjusted to align with the stripes, and the scanning direction is perpendicular to them. This configuration is beneficial for analyzing the adaptive adjustment process of the exposure time. The exposure time can be manually adjusted to an optimal value at the initial position. When adaptive control is not applied, the camera exposure time remains constant throughout the whole measurement process. When a darker-color stripe is scanned, the deviation in stripe quality parameter *E*(*t*) is reduced to a negative value. In this case, part of the laser stripes may become too dark to extract center points. Moreover, when the laser plane intersects the high reflective regions, the laser stripe may become overexposed, leading to a large positive deviation, as shown in Figure 6b.

When adaptive control is implemented, the exposure time can be adjusted during the same scanning process. This adjustment ensures that the deviation of stripe quality remains within the ideal range. The adaptive adjustment process of the stripe quality and camera exposure time over the entire measuring process is shown in Figure 6c. The camera’s exposure time increases in areas with low reflectivity and decreases at high-reflectivity regions. This ensures that *E*(*t*) is within an ideal range.

**Figure 7:**
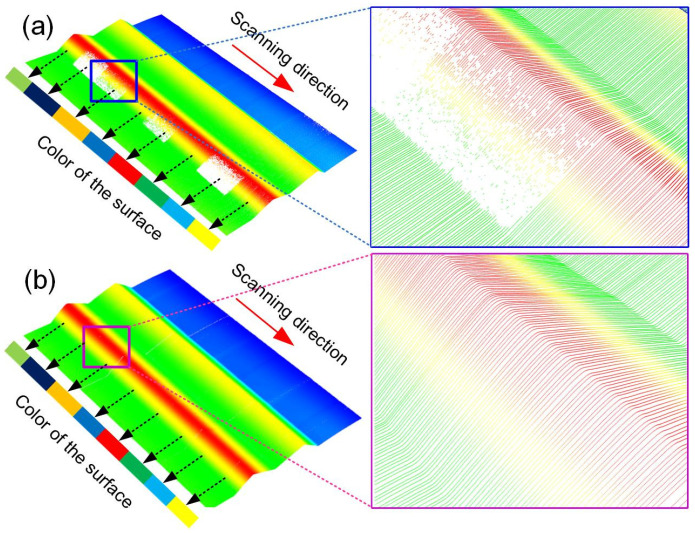
Measurement results of LSLS for the color stripe surface (**a**) without adaptive control and (**b**) with adaptive control.

The measurement results obtained without and with adaptive control are shown in Figure 7. Without adaptive control, the problem of underexposure exists in the low-reflectivity area, resulting in the loss of measurement data points, as shown in Figure 7a. When the adaptive control is employed, the measured result exhibits better integrity, as shown in Figure 7b. This further validates the superiority of the adaptive control method.

**Figure 8:**
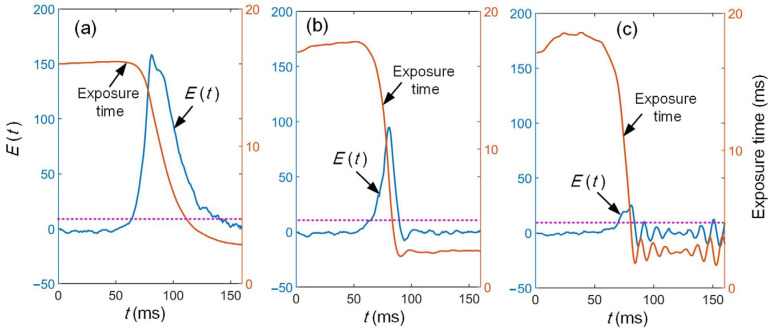
Changes in surface exposure time and stripe quality under different *K_p_* (**a**) *K_p_* = 0.2, (**b**) *K_p_* = 1.0, (**c**) *K_p_* = 4.0.

The proportional coefficient *K_p_* has a significant influence on the control performance. The adaptive adjustment process of exposure time is analyzed when the surface color changes abruptly (dark blue to beige). When *K_p_* = 0.2, the adjustment process is slow, as shown by Figure 8a. The exposure time cannot be tuned to the desired value in time, leading to an excessive deviation in quality parameters. It can also be seen that the fluctuation in the stripe quality has little influence on camera exposure time, resulting in a more stable exposure time. When *K_p_* is increased to 1.0, the maximum deviation of *E*(*t*) is rapidly reduced to the stable region, as shown in Figure 8b. The overshoot is only 2.7% and can quickly reach a stable state. As *K_p_* continues to increase, the maximum deviation decreases, yet not significantly, due to the oscillatory instability, as shown in Figure 8c.

To further investigate the relationship between the system performance and K*_p_*, the maximum deviations (MDs) are obtained and shown in Table 1. It can be seen that the MD decreases when *K_p_* increases. The stable time is computed with a tolerance of △ = 5%. When *K_p_* = 1.0, it also rapidly decreases to 25 ms. However, when *K_p_* is larger than 1.0, the stable time no longer decreases significantly. This is due to the instability of the system. Through the above experiments, it can be seen that the adaptive control system can successfully achieve the adaptive control according to the color of measured object. The stable time can be controlled within 30ms, even in the case of sudden color change. Thus, adaptive control can be realized during the scanning process.

### 3.3. Occlusion

**Figure 9:**
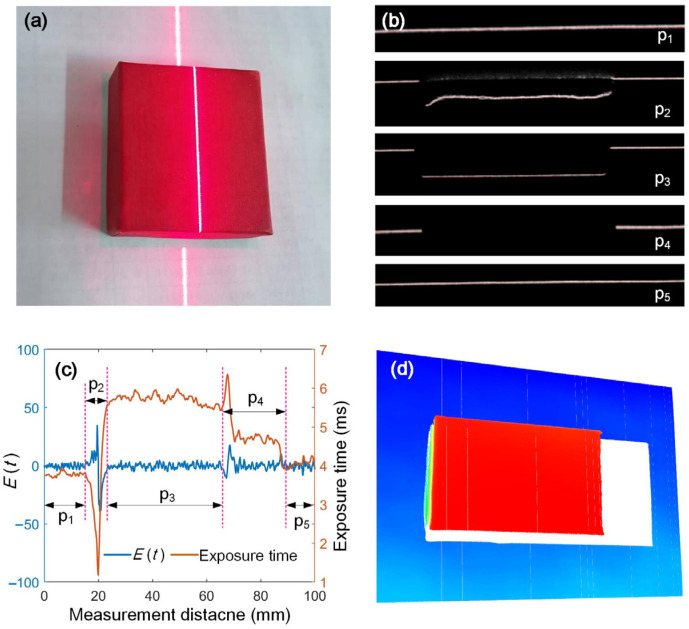
Scanning process and measurement results in the presence of laser stripe occlusion. (**a**) Measured object. (**b**) Images of the light stripe at different positions. (**c**) Changes in exposure time and quality of the stripe in measurement process. (**d**) Measurement results.

The measurement results are influenced not only by color, but also by geometry. When the object has a complex geometry, part of the stripe may be blocked by itself. To ensure the control stability, a coefficient of effective laser stripe, α, is introduced. The object is shown in Figure 9a. The whole measurement process can be divided into five stages: p_1_—the laser plane does not intersect the object, p_2_—the laser plane intersects the side of the object, p_3_—the laser plane intersects the top surface, p_4_—part of the stripe is blocked, and p_5_—the stripe moves out of the occlusion area. The typical stripe images corresponding to these stages are shown in Figure 9b.

The adaptive controlled exposure time during the scanning process is shown in Figure 9c. When the laser plane does not intersect the surface, the system automatically adjusts the exposure time to a stable value of approximately 4 ms. When the laser stripe is projected onto the side of the object, the light intensity reflected into the camera increases dramatically since this surface faces the camera. The stripe quality parameter also increases. To keep the quality parameter within the desired range, the control system adjusts the deviation by reducing the exposure time. When the laser stripe moves onto the top surface, the exposure time automatically increases due to the darker color. When part of the stripe is blocked, the control system can still modulate the quality parameters of the laser stripe. Therefore, the adaptive control has been realized for the whole scanning. Figure 9d is the measurement result which further validates the effectiveness of the control method.

### 3.4. Plane Measurement

**Figure 10:**
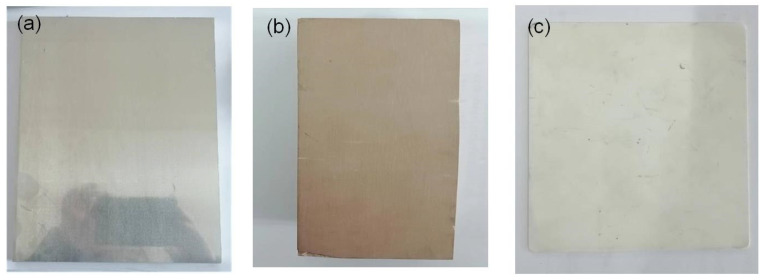
Flat surfaces of different materials (**a**) aluminum plane, (**b**) polyurethane plane, (**c**) ceramic plane.

To evaluate the performance of the control system, three typical planes with different materials and colors were measured. Figure 10a shows a precision aluminum plane, Figure 10b shows a polyurethane part after precision milling, and Figure 10c shows a ceramic plane (Al_2_O_3_). For conventional LSLSs, the camera’s exposure time is usually identical in the measurement of different surfaces or manually adjusted to a fixed value according to the material of measured surfaces.

**Figure 11:**
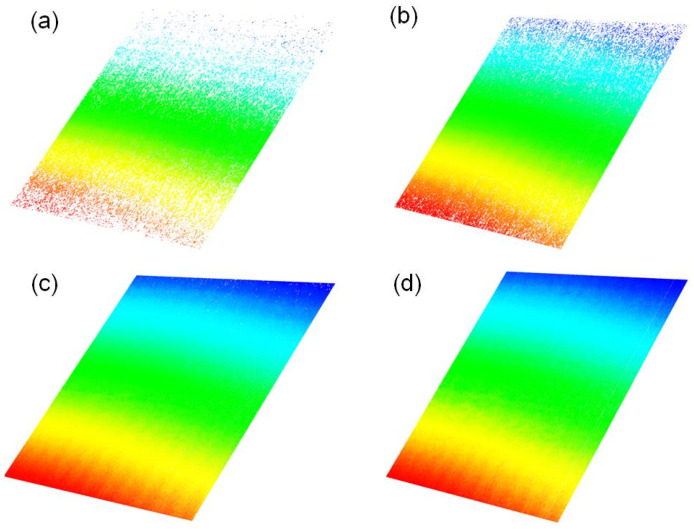
Measurement results of aluminum plane at different exposure times, (**a**) 1 ms, (**b**) 2 ms, (**c**) 6 ms, and (**d**) 10 ms.

The scanning processes of the aforementioned surfaces are carried out with different exposure times. The point cloud data of the aluminum plane are shown in Figure 11. When the exposure time is 1 ms, dense points can be obtained only in the center region. Massive points are lost at the edge due to underexposure. As the exposure time increases, the underexposed part of the laser stripe gradually reaches the threshold value, enabling the extraction of the stripe’s center.

**Figure 12:**
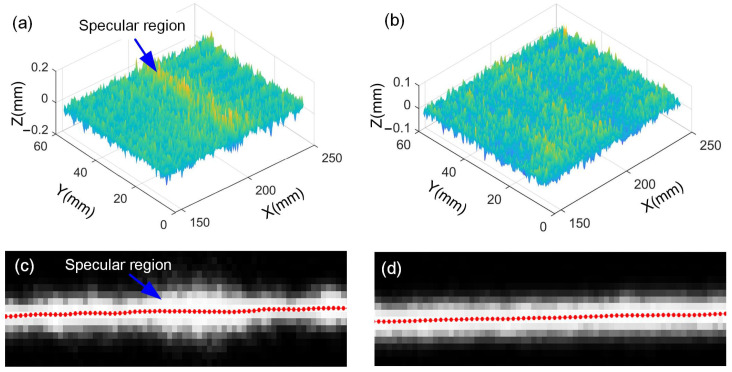
Measurement results and corresponding laser stripes: (**a**) aluminum plane, (**b**) ceramic plane, (**c**) laser stripe for aluminum plane, and (**d**) laser stripe for ceramic plane.

As the exposure time increases, the number of effective points can be significantly improved. For further analysis, the measurement results are corrected through plane fitting to remove the inclined component. The residual data are shown in Figure 12a,b. When adaptive control is implemented, partial images of the laser stripe are taken on the above three surfaces, and the center extraction results are shown in Figure 12c,d. It can be observed that for the aluminum plane, the middle region is overexposed, resulting in a large measurement error. For the ceramic plane, the laser stripes are more homogeneous, and a better center extraction result is achieved. Here, the width of the laser stripe with adaptive control ranges from 8 to 10 pixels. This is consistent with the conclusion in reference [31] that the uncertainty of the center extraction results achieves the smallest value when the stripe width ranges from 8 to 10 pixels.

The PV (Peak-to-Valley), AVR (average), and RMS (Root Mean Square) values of the three different measured surfaces were calculated at various exposure times, including the optimal time derived from adaptive control. The results are presented in Table 2, Table 3 and Table 4. For ceramic and aluminum planes, the PV value increases with the exposure time. The ceramic plane is white, and the laser stripe can reach a satisfied width at an exposure time of 1ms. On the other hand, the aluminum plane demonstrates a specular phenomenon, which makes part of laser stripe overexposed. The PV values also increase steadily with the increase in the exposure time.

### 3.5. Measurement and Analysis of Complex Surfaces

**Figure 13:**
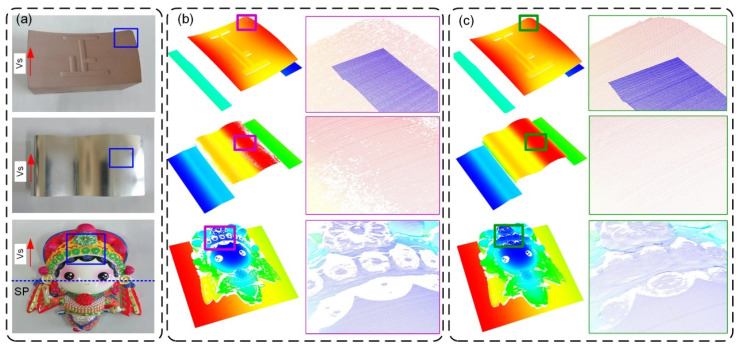
Typical complex surfaces and measurement results; (**a**) the measured object, (**b**) without adaptive control, and (**c**) with adaptive control.

To further validate the feasibility of the method, experiments were carried out on complex surfaces with different materials. Figure 13a shows three parts to be measured. The first one is a hyperboloid surface, on which several characters were carved. It was made of polyurethane, which has excellent diffuse reflection characteristics. The second one is an aluminum part which has a precision milled freeform surface. The third one is a ceramic part with rich colors. For the polyurethane plane in Figure 10b, it is easy to obtain ideal measurement results. However, for the complex surface in Figure 13a, the angle between the normal surface and the camera’s optical axis varies greatly. When this angle is small, the light entering the camera is intense, and overexposure is likely to occur. Conversely, when the angle is too large, the light entering the camera is weak, resulting in underexposure. This causes the loss of measurement points, as shown in Figure 13b. After measuring the polyurethane plane, the optimal exposure time is selected and is then directly applied to measure the aluminum and ceramic surfaces. In this situation, a large number of missing points can be observed, indicating that the optimal exposure time is not the same for different materials. However, when the adaptive control method is employed, the system can automatically adjust the exposure time based on the shape, surface material, roughness, and color of the measured surface. The improvement can be seen in Figure 13c.

For each intersection profile, the number of ideal center points should be equal to the column number of the stripe image. However, when the maximum gray value of a specific cross-section profile is less than the threshold *T_g_* = 70, the center point of this profile is not computed. This situation includes two possibilities. The cross-section profile is underexposed, or there is no stripe presented due to self-blocking. Otherwise, one center point can be computed for this column of the stripe. The ratio of effective points, *Q*_eff_, can be defined as

(6) $$Q_{\mathrm{eff}}=\frac{N_{\mathrm{eff}}}{V_{c}\cdot N_{\mathrm{val}}}\cdot 100\%\;,$$

where *N*_eff_ is the total number of effective points, *V_c_* is the column of each stripe image, and *N*_val_ is the number of stripe images. The ratio of effective points is presented in Table 5. It is evident that *Q*_eff_ can be notably enhanced for all scenarios by employing the adaptive control method.

**Figure 14:**
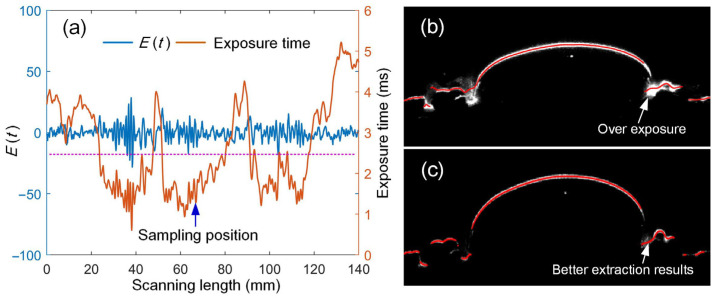
Adaptive measurement process and cross-sectional profile analysis. (**a**) Adaptive control process of exposure time during scanning (**b**) without adaptive control, and (**c**) with adaptive control.

Not only is the quantity of the point cloud important, so is the quality. The measured point cloud depends on the quality of the stripe images. Different objects (or even different areas of the same part) have diverse shapes and colors. If a fixed exposure time is adopted for the traditional sensors, the stripe image may experience underexposure or overexposure. Figure 14 shows a cross-sectional result during the scanning process of the ceramic part. Figure 14a illustrates the camera exposure time and stripe quality with adaptive control. It can be seen that the exposure time can be adjusted adaptively with the change in the surface, ensuring the stripe quality deviation within the ideal range. If a fixed exposure time (*t* = 2.5 ms) is employed, overexposure occurs when the laser stripe is scanned to this cross-section profile, as shown in Figure 14b. Overexposure leads to incorrect stripe center points. When adaptive control is applied, the system automatically reduces the camera’s exposure time, and the stripe center points are more accurate, as shown in Figure 14c. This further validates the necessity of the adaptive control method.

## 4. Conclusions

This paper proposed an adaptive control method for LSLMS. The method incorporates a quality evaluation strategy of stripe images based on global grayscale statistics. The relationship between the quality parameters of laser stripe and the camera exposure time is derived and analyzed. We find that the proposed stripe quality value grows linearly with the exposure time, which is beneficial for the adaptive control. In addition, the influence of the proportional coefficient on system performance is analyzed. The optimal performance can be achieved when the proportional coefficient is 1.0. With the control method, the camera’s exposure time is automatically adjusted according to the color of the measured surface. The laser stripe quality parameter can be controlled within the desired range and better results can be achieved. The measurement results of complex surfaces show that the adaptive control method improves the completeness and accuracy. It also has the advantages of simplicity and ease of use. In the future, intelligent control algorithms can be introduced to further improve the performance of the control system.

## Figures and Tables

**Table 1:** System performance under different proportional coefficients.

*K_p_*	0.2	0.6	1.0	1.6	2.0	3.0	4.0
MD	157.3	117.9	80.0	59.6	47.9	34.7	25.4
Stable time (ms)	76	36	25	26	23	20	18

**Table 2:** Measurement results of the aluminum plane (unit: mm).

Exposure Time	1 ms	2 ms	6 ms	10 ms	14 ms	18 ms	Optimal
PV	0.2608	0.2630	0.4494	0.6056	0.6265	0.7407	0.3112
AVR	0.0236	0.0225	0.0239	0.0326	0.0417	0.0502	0.0218
RMS	0.0297	0.0287	0.0313	0.0444	0.0563	0.0689	0.0283

**Table 3:** Measurement results of the polyurethane plane (unit: mm).

Exposure Time	1 ms	2 ms	6 ms	10 ms	14 ms	18 ms	Optimal
PV	0.3260	0.2202	0.1897	0.2257	0.2840	0.3213	0.1859
AVR	0.0263	0.0212	0.0182	0.0201	0.0257	0.0286	0.0181
RMS	0.0336	0.0267	0.0230	0.0253	0.0322	0.0361	0.0227

**Table 4:** Measurement results of the ceramic plane (unit: mm).

Exposure Time	1 ms	2 ms	6 ms	10 ms	14 ms	18 ms	Optimal
PV	0.1652	0.1866	0.2598	0.3225	0.3697	0.4875	0.1788
AVR	0.0144	0.0136	0.0221	0.0282	0.0384	0.0489	0.0126
RMS	0.0182	0.0172	0.0277	0.0355	0.0482	0.0609	0.0158

**Table 5:** Effect of adaptive control on the effective point ratio of the parts.

Parts with Different Materials	Polyurethane	Aluminum	Ceramic
Without adaptive control	79.2%	75.4%	68.6%
With adaptive control	82.9%	80.2%	70.5%

## Data Availability

The data are contained within the article.
